# Mouse Liver Sinusoidal Endothelium Eliminates HIV-Like Particles from Blood at a Rate of 100 Million per Minute by a Second-Order Kinetic Process

**DOI:** 10.3389/fimmu.2017.00035

**Published:** 2017-01-24

**Authors:** Jessica M. Mates, Zhili Yao, Alana M. Cheplowitz, Ozan Suer, Gary S. Phillips, Jesse J. Kwiek, Murugesan V. S. Rajaram, Jonghan Kim, John M. Robinson, Latha P. Ganesan, Clark L. Anderson

**Affiliations:** ^1^Departments of Internal Medicine, The Ohio State University, Columbus, OH, USA; ^2^Center for Biostatistics, Department of Biomedical Informatics, The Ohio State University, Columbus, OH, USA; ^3^Department of Microbiology, The Ohio State University, Columbus, OH, USA; ^4^Department of Microbial Infection and Immunity, The Ohio State University, Columbus, OH, USA; ^5^Department of Pharmaceutical Sciences, Northeastern University, Boston, MA, USA; ^6^Physiology and Cell Biology, The Ohio State University, Columbus, OH, USA

**Keywords:** liver sinusoidal endothelial cell, Kupffer cell, pinocytosis, endocytosis, clearance

## Abstract

We crafted human immunodeficiency virus (HIV)-like particles of diameter about 140 nm, which expressed two major HIV-1 proteins, namely, *env* and *gag* gene products, and used this reagent to simulate the rate of decay of HIV from the blood stream of BALB/c male mice. We found that most (~90%) of the particles were eliminated (cleared) from the blood by the liver sinusoidal endothelial cells (LSECs), the remainder from Kupffer cells; suggesting that LSECs are the major liver scavengers for HIV clearance from blood. Decay was rapid with kinetics suggesting second order with respect to particles, which infers dimerization of a putative receptor on LSEC. The number of HIV-like particles required for saturating the clearance mechanism was approximated. The capacity for elimination of blood-borne HIV-like particles by the sinusoid was 112 million particles per minute. Assuming that the sinusoid endothelial cells were about the size of glass-adherent macrophages, then elimination capacity was more than 540 particles per hour per endothelial cell.

## Non-Technical Summary

We have engineered a small particle that resembles a human immunodeficiency virus (HIV) in size and surface structure in order to learn how HIV travels in the blood circulation. We use the mouse as a model of the human. These particles, when infused into the blood stream, are removed from blood very rapidly, within minutes, mostly by a particular kind of cell that lines the blood vessels of the liver, a cell referred to as liver sinusoidal endothelial cell (LSEC) (ell-seck). The rate of removal from blood suggests complex details of the mechanism of removal. The capacity of LSEC to remove HIV-like particles is astonishingly high, namely, about 100 million HIV-like particles per minute. We can estimate that a single blood vessel-lining cell (LSEC) removes more than 500 particles per hour. Our findings have yet to be integrated into the understanding of the natural course of an HIV infection.

## Introduction

A readily apparent but poorly understood aspect of the innate immune response is the rapid and copious removal (or clearance) and subsequent degradation of blood-borne virus by the endothelium of the liver sinusoids (LSEC). This capacity of LSEC to remove virus is far more robust than like clearance by Kupffer cells (KC), which instead appear responsible mostly for the removal of larger particles such as bacteria and autologous cellular material (1). LSEC clear small particles other than virus in a similar manner, particles such as virus-like particles expressing polyoma virus proteins (2), small immune complexes made of ovalbumin and IgG antibody (3), lipopolysaccharide (4), and very likely other nanoparticles. The LSEC thus constitutes an outpost of the innate immune system with which cytokines have been associated only rarely (5). The rapid elimination of such particles has prompted the liver to be referred to in common parlance as the “garbage dump” of the body.

The molecular mechanism by which these small particles are cleared by LSEC is only beginning to be known: Fc receptors for IgG on LSEC are required, we have found, for the clearance from blood of small immune complexes (1, 3). Scavenger receptor B-1 (SRB1) binds and likely facilitates the removal of hepatitis virus C (HVC) from circulation (6, 7). More mechanistic details are needed.

We now continue these studies by characterizing carefully the rate and extent of removal of a virus-surrogate, i.e., a non-infectious HIV-like particle that expresses the translation products of the *env* and *gag* genes of HIV and thus has structural and antigenic characteristics suitable for recognition by the immune system, both innate and adaptive. This reagent will give us the opportunity later to study the effects of anti-HIV-1 antibody on virus or VLP removal by the LSEC (8).

## Results

We engineered a small particle the size of a virus (HIV-like particle) that consisted of a viral membrane derived from cultured HEK-293 cells and expressed features of HIV, namely, the HIV CXCR4 envelope protein (gp160) and the *gag* protein p24, but lacked nucleic acid and accessory proteins required for replication (M&M). We refer to the particle as an HIV-like particle. The diameter of the particles, measured by high-resolution analysis of Brownian diffusion, was ~140 ± 5 nm, mode ± SE, *n* = 43 (M&M). The concentration of suspensions of HIV-like particles we measured using a p24 ELISA.

**Figure 1:**
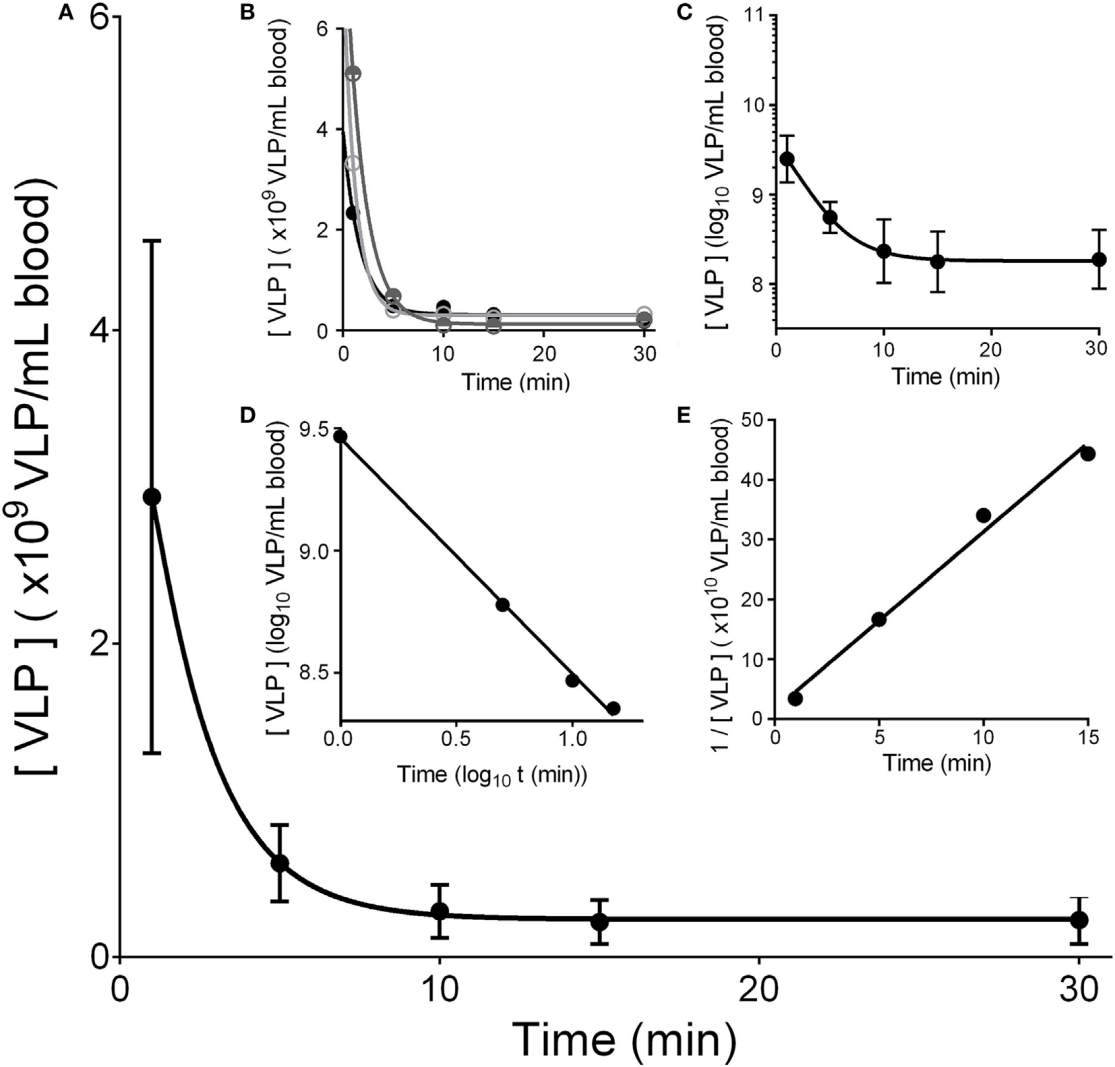
**HIV-like particles are cleared rapidly from murine circulation**. Approximately 2 × 10^10^ HIV-like particles were intravenously infused by tail vein. The concentration of particles remaining in blood of the suborbital plexus over time was estimated using a p24 ELISA. **(A)** Shows a plot of mean ± SD of the decay curves. The curve was drawn using asymmetric sigmoidal five parameters simulation to smooth the connection of data points. **(B)** Shows decay curves of three different mice illustrating the splay of the SD in **(A)**; one high, one mid-level, and one low. **(C)** Shows a log–linear plot of the data illustrating no straight line. **(D)** Plots the data in log–log fashion to reveal a straight line of pseudo second order kinetics. **(E)** Shows a reciprocal plot of the same data, showing also a straight line. The 30-min data points that did not fall on the straight line are not shown; they represent less than 3% of the dose. Each data point represents mean ± SD of 26 BALB/c wild-type mice.

To ascertain the rate of removal (or clearance) of HIV-like particles from the mouse circulation, we infused HIV-like particles intravenously and assessed their concentration in peripheral blood periodically over the course of 30 min (Figure 1). The decay curve was plotted in four ways. First, plotting simply using linear measurements for both the vertical axis showing concentration of HIV-like particles in blood (mean ± SD) versus the horizontal axis showing time in minutes, the decay curve showed two phases, a sharp drop followed by a lengthy near-plateau, with nearly all HIV-like particles (~97%) being cleared within 10 min. The second phase showed a plateau suggesting nil or negligible clearance. It represented only ~3% of the infused dose and thus was not included in further analysis (Figures 1D,E). As the SD of the first data point at 1 min was large, we additionally show decay curves of three mice that represent the SD splay (Figure 1B). Plotting the data in the conventional log-linear manner, the decay was curvilinear, not at all characteristic of the anticipated pseudo-first order reaction, which would show a straight line relationship (Figure 1C). However, showing the data as log–log and inverse linear–linear plots, we see straight lines (Figures 1D,E) (see Discussion).

**Figure 2:**
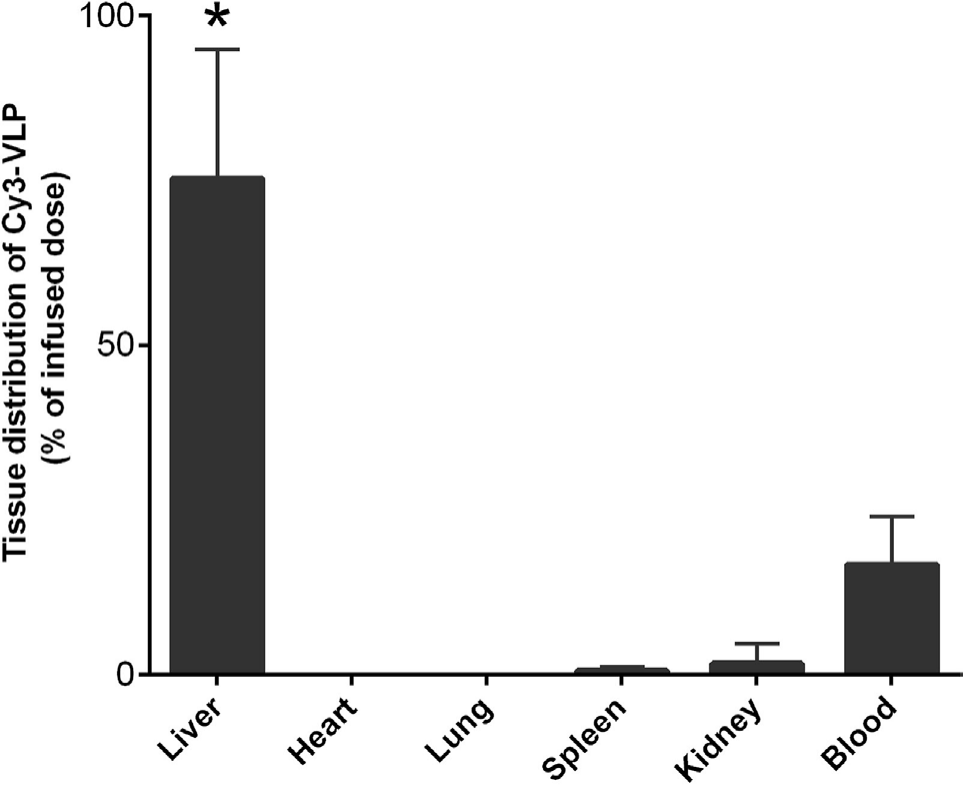
**The liver is the major organ clearing Cy3-HIV-like particles from blood**. Mice were infused with 10^10^ Cy3-human immunodeficiency virus-like particles and, after 10 min of infusion, the Cy3 fluorescence was quantified in various organs as described in M&M. The bar graph expresses the percentage means and SDs of total Cy3 fluorescence that was recovered from the six organs studied. The asterisk represents data points where the *p*-Values were determined to be less than 0.05 using Student’s *t*-test.

We found experimentally that the liver is the major organ clearing Cy3-HIV-like particles from blood (Figure 2): the organ distribution experiment showed that, in 10 min, the majority of total recovered Cy3-HIV-like particles was associated with liver (~80%), whereas less than 2% of the total recovered dose associated with spleen and kidney. The fraction of the dose of HIV-like particles that associates with blood shown in the bar graph is not statistical significantly different (*p* > 0.05) from the amount that we have estimated in the clearance curves using the unlabeled HIV-like particles (Figure 1). In addition, an experiment comparing the clearance of unlabeled HIV-like particles with that of the Cy3-HIV-like particles (data not shown) suggests that the clearance kinetics were not significantly different, assuring that the labeling of HIV-like particles with Cy3 did not change the clearance property of the particle. These data, indicating strongly that the liver is the major organ clearing circulating HIV-like particles, are in concert with several published studies showing liver to be the major site of removal of a variety of blood-borne viruses and virus-like particles, with minimal uptake occurring in the lung, spleen, and kidney (1, 2, 9–12).

**Figure 3:**
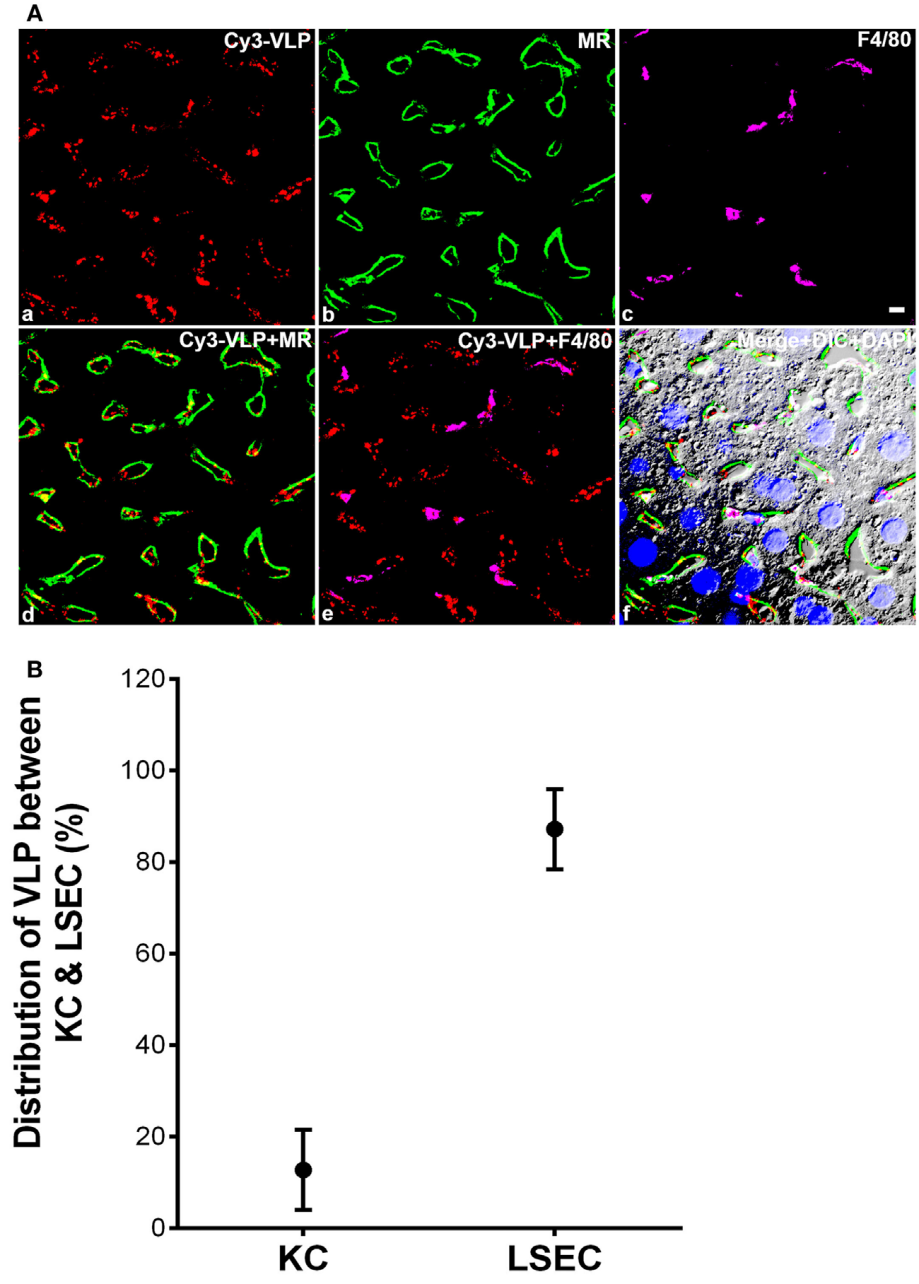
**The majority of human immunodeficiency virus (HIV)-like particles cleared by liver is localized to the liver sinusoidal endothelial cell (LSEC)**. **(A)** Four-color fluorescence microscopic images of 5 µm liver sections, 3 min after intravenous infusion of 2 × 10^10^ Cy3-HIV-like particles. (a) Red puncta show Cy3-HIV-like particles. (b) rabbit anti-mannose receptor (CD206) labeling of LSEC shown in green. (c) rat mab F4/80 labeling of KC shown in magenta. (d) Cy3-VLP (red) merged with LSEC marker. (e) Cy3-HIV-like particles (red) merged with KC marker. (f) Merged image showing Cy3-HIV-like particles (red), LSEC marker (green), and KC marker (magenta) plus DIC and DAPI staining of the nuclei (blue). Panels shown are representative of 160 images from three different mice. The scale bar in panel (c) signifies 5 µm. **(B)** Quantified association of HIV-like particles with cells of the liver. All HIV-like particles in the liver were associated with either LSEC or KC, indicating no association with hepatocytes. The total HIV-like particles was calculated as the pixel area × mean fluorescence intensity (red). HIV-like particles associated with the KC was subtracted from the total HIV-like particle to calculate LSEC association of HIV-like particle. The graph represents the mean ± SD of KC- and LSEC-association within liver. One hundred sixty images, roughly 50 from each of the three mice, were examined. The area totaled 30 mm^2^ of sectioned liver tissue.

Also, 3 min after the intravenous infusion of a dose, we determined the cellular localization within the liver of fluor-labeled HIV-like particles by examining 5 µm sections of mouse liver using immunofluorescence confocal microscopy, distinguishing LSEC with fluor-tagged anti-mannose receptor antibody, and KC with fluor-tagged anti-macrophage antibody. Visual microscopic inspection indicated that the HIV-like particles were situated on or in sinusoidal cells and were absent from the lumens of cross-sectioned sinusoids. The particles were much more abundantly associated with LSEC than with KC (Figure 3A). We have earlier illustrated the validity of these markers (1, 3). Substantiating this disparity quantitatively by estimating pixel fluorescence numbers and intensity, we found that 88% of HIV-like particles associated with LSEC while 12% associated with KC (Figure 3B). HIV-like particles were not found associating with hepatocytes. Additionally, the HIV-like particles associated with KC appeared aggregated while virus-like particles associated with LSEC appeared as fine puncta.

To ask whether liver uptake of HIV-like particles is saturable, we observed in Figure 1 that nearly the entire dose (2 × 10^10^ HIV-like particles) was removed from blood in 10 min (~97%), which suggests that the clearance mechanism was not saturated. Further, increasing the dose to 4 × 10^10^ did not saturate the clearance mechanism as, again, nearly the entire dose was cleared in 10 min. Re-evaluating a published study of ours showing decay curves of human recombinant adenovirus (rAd5) in mice, saturation was not achievable with an intravenous dose of 1.6 × 10^11^ rAd5 particles (1). Specifically, plotting vertically on a log scale three doses of rAd5 over a 2-log range versus, on the horizontal axis, the number of rAd5 removed in 10 min showed a straight line with a positive slope, indicating that the uptake mechanism was not saturated [curve not shown; data presented in Figure 1A of our paper (1)]. For technical reasons, we were unable to achieve higher doses of HIV-like particles; thus, with our present strategy failing to show saturation of the uptake mechanism, we can only assume that we are near saturation. For practical purposes, we assume, then, that a saturation dose is near 2 × 10^10^.

**Figure 4:**
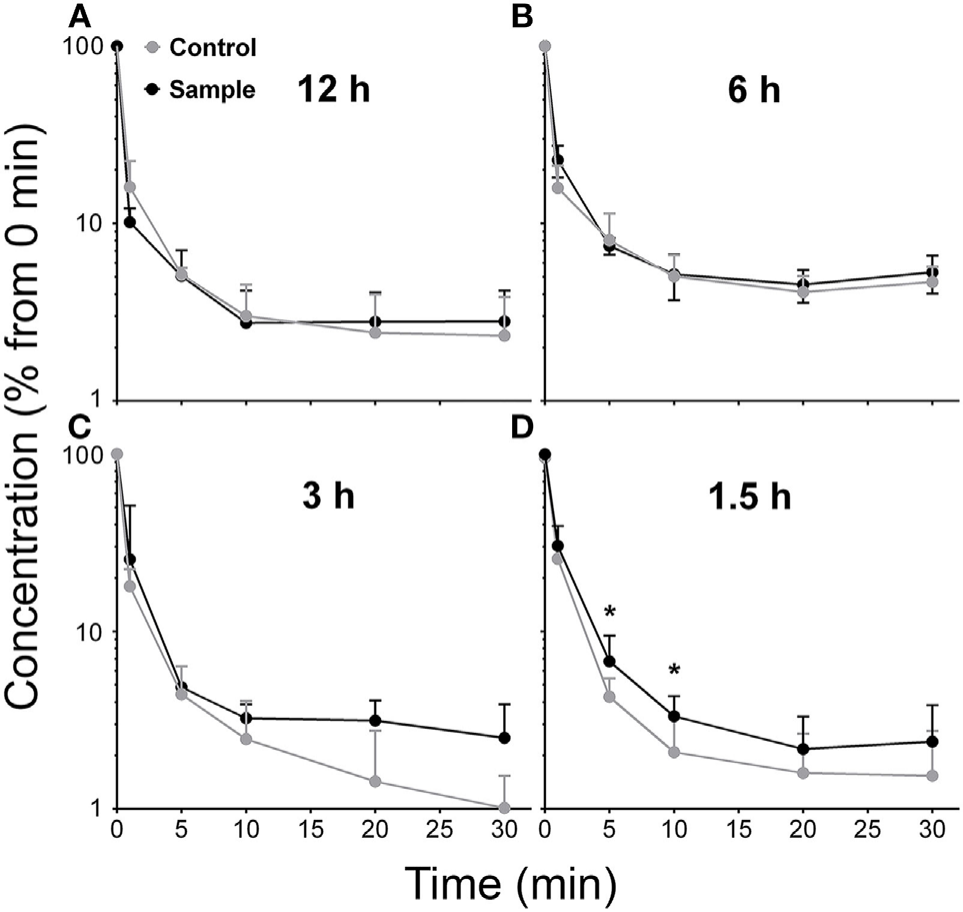
**Clearance capacity of human immunodeficiency virus (HIV)-like particles is fully recovered in about 3 h**. Mice were infused intravenously with 2 × 10^10^ HIV-like particles, allowed to recover for the indicated time (1.5, 3, 6, and 12 h) and then were infused with an additional bolus of 2 × 10^10^ HIV-like particles. The blood concentration of HIV-like particles was determined as described in Figure 1; the (sample) curves are to be compared with decay curves performed simultaneously on mice that had not received the initial dose of HIV-like particles (control). Each data point represents mean ± SD of several BALB/c wild-type mice. The number of animals used at each time period 1.5, 3, 6, and 12 h was 6, 3, 3, and 4, respectively. The raw data were log_10_-transformed prior to running the random-effects linear regression model, described in M&M. The corresponding points on the two curves in panels (12, 6, and 3 h) were not statistically different, even the apparently divergent points at 3 h. However, the corresponding points on the curves in panel (1.5 h) were statistically significantly different at the 5 and 10 min points, but not at the others. Thus, the recovery time was estimated to be between 1.5 and 3 h.

Proceeding, we reasoned that the removal from circulation of a large dose of HIV-like particle (2 × 10^10^) would leave the liver unable to remove a second dose as efficiently as the first; i.e., the liver may require time to recover its native removal ability. To calculate this refractory period that follows a first dose, we infused a second dose at varying times (1.5–12 h) after the first dose and plotted decay curves of the second dose, comparing the two decay curves performed simultaneously, the second with the first. As measures of differences both in the extent and rate of decay, we compared the numbers of HIV-like particle cleared at every point on the two curves, experimental, and control (Figure 4).

The decay curves performed 12 and 6 h after a first dose were fully superimposable on the initial control decay curves and were not statistically significantly different from the control decay curves (Figures 4A,B). The decay curve at 3 h after the initial dose appeared to show disparity of later points, but the experimental and control points throughout the entire curves were not statistically significantly different (Figure 4C). However, at 1.5 h after the initial dose, the decay curve plateaued earlier than the control curve (Figure 4D); the two curves were statistically and significantly different at the 5 and 10 min points (asterisks).

## Discussion

The HIV-like particle used in this study was crafted to express multiple copies of two major HIV gene products, the surface-expressed gp160 *env* protein for eventual use as an antibody target, and the *gag* protein p24 for use with an immunoassay.

We have learned from these data that the rate and extent of removal of blood-borne HIV-like particle in the mouse shows a biphasic decay curve with a very rapid and extensive first phase and a negligible second phase. Thus, decay is similar to the removal of blood-borne virus as studied by us and others [see our paper (1) for brief review]. However, we perceive a distinct difference from the decay of Ad5. By analogy with standard chemical reactions, the straight lines of the graphs of Figures 1D,E are characteristic of pseudo-second order reactions with respect to HIV-like particle concentrations, assuming a constant concentration of the sinusoidal binding site for HIV-like particles. In standard chemical parlance, the reaction is represented as 2A + B → AAB. In contrast, adenovirus clearance using data from an earlier study plotted in log-linear fashion showed a straight line characteristic of a pseudo first order reaction (1).

We can only speculate what the apparent second order of VLP decay might mean. At face value, the data would suggest that two HIV-like particles are interacting as a unit with an LSEC target. We know of no reason why HIV-like particles should dimerize, and in fact, according to our Brownian motion detector, the HIV-like particles were monodisperse. Whether they dimerize in blood, we have no way of determining. It is also possible that the binding site on LSEC is a dimer, although we know of no evidence that CD4 or L-SIGN, both known to be expressed on LSEC (13, 14), dimerize. However, there is precedence for LSEC to express dimeric receptors; i.e., SRB-1, a receptor for HDL and HCV envelope protein E2 expressed on LSEC (6, 7, 15), is known to dimerize and oligomerize (16). What precisely second order means in the case of our Figure 1 data awaits additional study.

We attempted to saturate the clearance mechanism of LSEC but were not at all successful. Doses of HIV-like particles as high as practicable, 4 × 10^10^, were cleared nearly completely (97%) in 10 min, indicating that saturation had not yet been reached. Nor did we reach a saturating dose in our prior studies of human adenovirus clearance where doses equally high, 1.6 × 10^11^, were infused (1). For technical reasons, we were unable to study higher doses; thus, we assume that saturation is 2 × 10^10^.

Failing to find a saturating dose, we nevertheless were able to estimate a recovery time by examining closely the shape of the decay curves after a second dose of HIV-like particles. The decay curve of a second dose 1.5 h after the first was clearly different from the control, indicating, albeit tenuously, that the clearance mechanism was still recovering at 1.5 h. At 3 h, moreover, the decay curve looked abnormal in that it diverged from the control curve, but statistically it was no different than the control curve. By 6 h, however, the clearance mechanism had returned to superimposable on the normal curve and remained superimposable at 12 h. We have not calculated half-lives because half-life is a function of dose in second order reactions; we did not vary the dose substantially. For practical purposes, we arbitrarily let recovery time be 3 h.

Assuming that the greatest dose used in our experiments (2 × 10^10^) is close to the saturation dose (S), and that the recovery time (R) is 3 h, we can then calculate the capacity (C) of the liver to remove HIV-like particles from circulation in an on-going, continuous fashion. We define capacity in units of particles per day as the saturation dose divided by the recovery time R (C = S/R). Specifically, every 3 h, 2 × 10^10^ HIV-like particles were removed from circulation. The capacity for clearance, therefore, is 6.7 × 10^9^ (2 × 10^10^/3) HIV-like particles per hour per mouse.

Is this a biologically realistic number? Capacity expressed as functions of hourly or daily clearance are numbers too large for us to conceptualize. However, converting to clearance per minute gives an everyday number that most workers will appreciate; i.e., 112 million HIV-like particles per minute (6.7 × 10^9^/60 min). This number sounds realistic and substantial. In fact, the number sounds remarkably potent when compared to a lethal dose of an arbitrarily chosen widely known virus (influenza virus) which, given intratracheally, has been shown to be on the order of 10^4^ virus particles (personal communication, Ian Davis, The Ohio State University).

Can we put the clearance capacity into perspective by asking how large is the sinusoid area responsible for clearing 112 million particles/min? Calculating from the rat sinusoid area (17), assuming that mouse liver weight is 1 g, we find that the surface area of the mouse sinusoidal network is 5.8 × 10^10^ μm^2^, or roughly half the area of the face of a tennis racquet.^1^ Another way of rendering realistic the magnitude of the sinusoidal surface area is by thinking of it as covered by confluent adherent macrophages. Assuming that the area of an adherent macrophage is 4.5 × 10^3^ μm^2^ (Figure 5 of publication PMC3488130), then the mouse sinusoid would be covered by 13 million confluent glass-adherent macrophages. Assuming further that the area of the adherent macrophage is about the same as a sinusoidal endothelial cell, then we find that 540 HIV-like particles are cleared per LSEC per hour (calculations in Supplementary Material).

What is the fate of the HIV-like particles once cleared from blood and bound to the LSEC? We interpret our data to indicate that HIV-like particles, cleared from blood, associate mostly (88%) with LSEC, to a small extent with KC (12%), and not at all with hepatocytes. At 3 min after infusion (Figure 3), they appear on or within the endothelium but not luminal as evidenced by their absence in luminal cross-sections. At 10 min after infusion, we find virtually no HIV-like particles associating with LSEC or KC (data not shown). We presume, based on the fate of immune complexes (18, 19), that most bound particles are internalized and degraded, although we have not yet embarked on a formal study of HIV-like particles. The literature indicates that endocytosed particles of many sorts are degraded (2, 11); other studies suggest that a fraction of the endocytosed particles is degraded and a fraction is expressed back onto the surface of the endocytosing cell (18, 20). The general impression that endocytosed particles are disposed of quickly is consistent with the novelty of our suggestion; virus particles internalized by LSEC are not ordinarily described during the course of virus infections. In our recent study of LPS clearance from blood, we found no evidence that cleared LPS moved from LSEC to KC (4). A systematic study of the fate of cleared particles is needed.

A remarkable implication of this robust clearance capacity is that clearance may be fast enough to avoid detection in the blood by culture or nucleic acid assay while allowing hematogenous spread of infection. It would follow that blood cultures and nucleic acid assays would not become positive until the clearance capacity of the liver is saturated. These implications are testable.

Further, we would propose that two different viruses may compete for a single clearance mechanism, although to date no such evidence is available. Such studies will require a keener analysis of the clearance mechanism and its discrimination among various bound particles. In support of this speculation, it has been known for decades that small particles in blood such as thorotrast will block the LSEC uptake of virus (10).

Finally, this astonishingly rapid and robust removal of blood-borne virus would appear to have been overlooked by all but a few modern and early biologists studying virus turnover during infection (10–12, 21). To us, this mechanism appears to be a subdivision of the innate immune system that has received little attention but might very well be of immense value to the organism. Many additional consequences of our observation, we anticipate, will become clear with appropriate study.

As a postscript, we point out that the rapidity of particle clearance described herein is remarkably similar to the classical “distribution” phase of the decay curves of protein and drug clearance from blood, a phenomenon well described in the pharmacokinetics literature.^2^ However, beyond rapidity, the similarity stops. Virus leaving the plasma compartment bound to the LSEC surface does not appear to be in equilibrium with the plasma compartment, which by definition it must be if the rapid portion of decay is to be considered “distribution” of kinetic decay. We imagine that the rapidity seen in our studies is simply quick clearance on a pathway toward ultimate degradation or processing by the sinusoidal cells.

## Materials and Methods

### Animals

Wild-type male BALB/c mice of age 10–15 weeks were obtained from Taconic Biosciences. All studies were performed in accordance with appropriate guidelines and were approved by The Ohio State University Institutional Animal Care and Use Committee. All *in vivo* mouse procedures were performed under Isoflurane anesthesia.

### Plasmids

The pGag-EGFP plasmid (NIHARP cat #11468) used to prepare HIV-1 VLP, which directs Rev-independent expression of HIV-1 Gag-EGFP fusion protein (tier 1 clade B) to form VLP, was obtained from Dr. Marilyn Resh through the NIH AIDS Reagent Program, Division of AIDS, NIAID, NIH. The pGag-EGFP plasmid was constructed by cloning Gag from pCMV55M1-10 (22) into the pEGFP-N1 plasmid (Clontech) (23). Plasmid DNA was amplified in *Escherichia coli* DH5α; DH5α-containing pGag plasmid was grown in LB medium supplemented with 25 µg/mL kanamycin. The pHXB2 envelope plasmid (NIHARP cat#1069), containing HXB2 gp160 under an SV40 promoter, was obtained from Dr. Kathleen Page and Dr. Dan Littman through the NIH AIDS Reagent Program, Division of AIDS, NIAID, NIH. Plasmid DNA was amplified in *E. coli* DH5α; DH5α-containing pHXB2 env plasmid was grown in LB medium supplemented with 50 µg/mL ampicillin. Plasmid purification used the BenchPro 2100 Plasmid Purification System (Invitrogen).

### Preparation of VLP-Containing HXB2 env

VLP were propagated using human embryonic kidney, 293T cells (ATCC). Cells were maintained in Dulbecco’s Modified Eagle medium with 10% Fetal Bovine Serum. VLP-containing HXB2 envelope was produced by transient transfection of HEK 293T cells with pGag-EGFP and pHXB2 env using Lipofectamine 2000 transfection reagent (Life Technologies). Also, 10^7^ cells in T175 flasks were transfected with 30 µg HXB2 envelope, 60 µg pGag-EGFP, and 360 µL Lipofectamine 2000 transfection reagent in serum/antibiotic-free medium. After 3–4 h of incubation at 37°C, the culture medium was replaced with DMEM + 10% FBS. VLP-containing supernatant was collected 72 h after transfection and clarified by centrifugation at 2,000 × *g* for 10 min. Clarified supernatant was further purified of cellular debris by 0.45 µm filtration. Purification of assembled VLP was completed by ultracentrifugation through a 20% sucrose pad at 122,000 × *g* for 2 h at 4°C. The VLP pellet was resuspended in filtered PBS.

### Quantification of VLP

Two methods were used to quantify VLP concentrations, p24 ELISA and Nanoparticle Tracking Analysis (NTA). p24, a viral protein component Gag, was used to determine VLP concentration with the commercially available Zeptometrix p24 ELISA kit in accordance with the manufacturer’s instructions. The lower limit of detectability in the assay was 10^7^–2 × 10^7^ particles/mL. Additionally, NTA was performed using a Nanosight NS300 (Malvern). VLP samples resuspended in filtered PBS were diluted to approximately 10^8^–10^9^ particles/mL; 1 mL of diluted VLP sample was injected into the Nanosight apparatus. Nanosight NTA 3.0 software was used to analyze nanoparticle tracking data. Five individual videos ranging from 30 to 60 s each were recorded and analyzed based on the VLP Brownian motion at room temperature. NTA analysis determined both particle size and concentration of VLP per milliliter. A ratio of p24 concentration (picograms per milliliter) to NTA (VLP per milliliter) was calculated for each VLP preparation.

### Cy3 Labeling of VLP

VLP in PBS, pH 7.4, at a concentration of 10^12^ VLP/mL was adjusted to pH 9.4 by the addition of 0.5 M sodium carbonate bicarbonate. The Cy3 monoreactive dye pack (Amersham) was dissolved in 1 mL of VLP solution, pH 9.4, and incubated at room temperature for 30 min with constant stirring. The addition of 0.2% glycine stopped the labeling reaction, and the Cy3-labeled VLP was dialyzed against two changes of PBS, pH 7.4, at 4°C for 18 h. The efficiency of Cy3-conjugation was assessed by comparing the protein concentration (micrograms per microliter) of the dialyzed Cy3-conjugated VLP with the concentration of Cy3 (picomoles per microliter). The Cy3 concentration was converted to micrograms per microliter using the formula weight; the dye to protein ratio of Cy3-conjugated VLP was 0.02.

### Immunofluorescence

BALB/c mice 11–14 weeks old were intravenously infused with 2 × 10^10^ Cy3-labeled VLP in PBS, pH 7.4. BALB/c livers were excised, cut into ~5 mm pieces, and fixed in 4% paraformaldehyde-PBS for 2 h at room temperature. Fixed tissue was washed with PBS and saturated with 20% sucrose-PBS overnight at 4°C. Upon sucrose saturation, tissue was embedded in tissue-freezing medium and stored at −80°C. Fixed and frozen tissue was sectioned at 5 µm thickness by Cyrostat sectioning and collected on Superfrost microscope slides. Tissue sections were rehydrated, blocked in 5% milk blocking solution for 1 h, and then incubated with primary antibodies in 5% blocking solution overnight at 4°C. Unconjugated primary antibodies were visualized using a 1:200 dilution of fluor-tagged secondary antibodies in 5% blocking buffer for 1 h at room temperature. DAPI staining was executed for 10 min and then tissue sections were mounted under coverslips with Prolong Gold Solution (Invitrogen). Isotype controls along with secondary antibodies were used to assess primary and secondary immunostaining.

Primary antibodies included rabbit polyclonal IgG anti-CD206 mannose receptor (Santa Cruz) and rat monoclonal IgG anti-F4/80 (Abserotec). Secondary antibodies from Invitrogen included Alexa 488-conjugated goat anti rabbit IgG and Alexa 680-conjugated goat-anti rat IgG.

Images were acquired using 405, 488, Cy3, and 680 laser settings on an Olympus Fluo View 1000 Laser Scanning Confocal microscope with a spectral detection system designed for finer separation of fluorochromes (FV 1000 Spectral).

### Quantification of HIV-Like Particles in Various Organs

Approximately 10^10^ Cy3-labeled HIV-like particles were infused *via* the retro–orbital plexus of anesthetized mice and were sacrificed at 10 min. The mice were bled of ~20 μL *via* the retro–orbital plexus, and organs (liver, kidney, lung, spleen, and heart) were removed and weighed. The weighed portions of the organs were homogenized and lysed with organ lysis buffer (0.1% SDS, 10 mM Tris, pH 7.4, and 1 mM EDTA) (3). The Cy3 fluorescence in organ lysates and blood was measured using fluorimeter (2300 Enspire multimode reader). The amount of Cy3 fluorescence associated with each organ was estimated by factoring the total organ weight and after subtracting both the blood volume of that particular organ (24) and the background organ fluorescence from un-infused age matched mice.

### Quantification of the Association of Cy3-Labeled VLP with Liver Cells

Based on 4-color immunofluorescence analysis of liver sections, we determined that Cy3-labeled VLP associated with LSEC and KC, but not hepatocytes or large vessel endothelium. The association of VLP with LSEC and KC was quantified with Image J software as described previously in our work (1). Briefly, quantification was determined in a three-step process: (1) image threshold was adjusted to account for background intensity not associated with Cy3 VLP, and the total Cy3 fluorescence intensity was recorded for the image; (2) each Kupffer cell (KC) within the image, identified by anti-F4/80 staining, was partitioned, and the fluorescence intensity of Cy3 associated with individual KC was recorded; the VLP intensity associated with individual KC was summed to total KC-associated VLP; and (3) the total KC-associated VLP intensity was subtracted from the total VLP intensity of the entire image to give the total LSEC-associated VLP intensity. KC and LSEC association was averaged for a total of 160 technical replicates of the images over the three mice. Within each mouse, the technical replicates were averaged producing three KC and three LSEC associations. The mean and SD of the three KC- and the three LSEC-association observations are presented and no statistical testing was done since these observations are not independent of each other.

### Clearance of VLP from the Bloodstream

Clearance is defined as elimination from blood. BALB/c male mice at age 14 weeks were infused intravenously *via* tail vein with 2 × 10^10^ VLP in PBS, pH 7.4. After infusion, 20 µL of blood was obtained *via* the retro–orbital plexus at 1, 5, 10, 20 (or 15), and 30 min using heparinized capillary tubes. The calculation of VLP concentration at the time of infusion (time 0) was based on the mouse weight and assumed total blood volume of ~2.58 mL/25 g mouse (25). Blood obtained at each time point was diluted and VLP were quantified using p24 ELISA (Zeptometrix) per the manufacturer’s instructions. The concentrations of p24 in picograms per milliliter were converted to VLP per milliliter using the calculated conversion rate between pictograms per milliliter and NTA. Clearance kinetics were plotted as concentrations of VLP per milliliter of blood versus time. The curve was biphasic with a fast phase, an inflection at about 10 min, at which most of the VLP had been cleared (97%), and a second phase, a virtual plateau.

### Statistics

The following method was used for the recovery time experiments. VLP concentration was log_10_-transformed, and all analyses were run on the transformed values. A random-effects linear regression model where log_10_-transformed VLP concentration was the dependent variable while group (study versus control mice), recovery period (1.5, 3, 6, 12, and 24 h), test time [0, 1, 5, 10, 20 (or 15), and 30 min], and all two-way interactions were included in the model as independent categorical variables. Random-effects regression was used due to the longitudinal nature of the observations where the outcomes (log_10_ VPL concentration) were nested within specific mice over time. Additionally, this method uses all of the results from all of the mice used in the study. After running the random-effects linear regression model, linear contrast statements were used to estimate differences between study and control mice for specific test times and recovery periods of interest. In addition to these differences, the 95% confidence interval of the differences and the *p*-Values are also produced by the linear contrast statements. All analyses were run using Stata 14.1, StataCorp LP, College Station, TX, USA.

## Author Contributions

Conceived and designed the experiments: CA and LG. Performed the experiments: JM, ZY, AC, OS, MR, and GP Analyzed the data: JM, ZY, AC, OS, GP, JK, MR, JK, JR, LG, and CA. Contributed reagents/materials/analysis tools: JK and GP. Wrote the paper: CA.

## Conflict of Interest Statement

The authors declare that the research was conducted in the absence of any commercial or financial relationships that could be construed as a potential conflict of interest.

## Footnotes

^1^http://officialtennisrules.com/official-dimensions-for-tennis-rackets/.

^2^http://sepia.unil.ch/pharmacology/?id=93.

## Abbreviations

LSECs, liver sinusoidal endothelial cells; KC, Kupffer cells; DIC, differential interference contrast; IF, immunofluorescence; RFI, relative fluorescence intensity; VLP, HIV-like particle.
