# Supplementary material for: Mouse Liver Sinusoidal Endothelium Eliminates HIV-Like Particles from Blood at a Rate of 100 Million per Minute by a Second-Order Kinetic Process
Source: Front Immunol. 2017 Jan 24;8:35. doi: 10.3389/fimmu.2017.00035 (PMC5256111; doi:10.3389/fimmu.2017.00035)
Supplement: Supplementary file 1 [file Presentation_1.pdf]

Supplemental text

TITLE: Mouse liver sinusoidal endothelium eliminates HIV-like particles from blood at a rate of 100 million per minute by a second order kinetic process.

AUTHORS: Jessica M. Mates[1], Zhili Yao[1], Alana M. Cheplowitz[1], Ozan Suer[1], Gary S. Phillips[2], Jesse J. Kwiek[3], Murugesan V. S. Rajaram[4], Jonghan Kim[5], John M. Robinson[6], Latha P. Ganesan[1], and Clark L. Anderson[1]

AFFILIATIONS: [1]Departments of Internal Medicine, [2]Center for Biostatistics, Department of Biomedical Informatics, [3]Department of Microbiology, [4]Department of Microbial Infection and Immunity, [6]Physiology and Cell Biology, The Ohio State University, Columbus, OH 43210; [5]Department of Pharmaceutical Sciences, Northeastern University, Boston, MA 02115.

CORRESPONDING AUTHOR: Clark. L. Anderson, The Ohio State University, 110 HLRI, 483 West Twelfth Avenue, Columbus, OH 43210. Phone: (614) 247-7650; Fax: (614) 247-7669; email: anderson.48@osu.edu; www.andersonlab.com

Outline for calculation of area of sinusoidal membrane, number of LSEC / mouse, and number of particles / LSEC.

1. Rat liver endothelial cell (LSEC) surface area is 0.116 m^2/cm^3 according to Table 3 of a stereological analysis [1] and assuming $1cm^3$ = 1g liver.

2. Divide by 2 to obtain the area of the luminal surface of rat LSEC alone. Half is 0.058 m^2 / g because sinusoid endothelium has two plasma membrane surfaces, luminal and abluminal.

3. Convert area in m^2 to μm^2. ($1\ m^2 = 10^{12}\ \mu m^2$) So, area rat LSEC luminal plasma membrane = $0.058\ x10^{12}\ \mu m^2$ / g liver or = $5.8\ x10^{10}\ \mu m^2/g$

4. Assume membrane area/g of rat liver is same as membrane area/g of mouse liver. Our mouse livers weigh 1 g

5. So, area of mouse liver sinusoidal endothelium membrane is $5.8x10^{10}\ \mu m^2$

6. or roughly half the area of the face of a tennis racquet (http://officialtennisrules.com/official-dimensions-for-tennis-rackets/)

7. If surface area of glass-adherent macrophages (mac) is $4.5\ x10^3\ \mu m^2$ (Fig 6 of http://www.ncbi.nlm.nih.gov/pmc/articles/PMC3488130/)

8. Then the number of mac that would line a mouse sinusoid would be $5.8x10^{10}$/ $4.5x10^3 = 1.3x10^7$, or 13 million macs/sinusoid

9. If assume area of mac and LSEC are roughly the same, then the mouse liver sinusoid is lined with 13 million LSEC.

10. Particles cleared per unit time per mouse: Every 3 hours $2x10^{10}$ HIV-like particles were removed from circulation. The capacity for clearance, therefore, is $6.7x10^{9}$ ($2x10^{10}$ / 3), or 6700 million HIV-like particles per hour per mouse, or 6700/60=112 million per min/mouse.

11. If particles cleared by liver is 112 million/min, then how many per LSEC? 112 million/min divided by 13 million LSEC = 112/13 = 9 particle/minute/LSEC. Since 60 min/hour, then 540 particles/hour/LSEC.

Reference List

[1] A. Blouin, R.P. Bolender, E.R. Weibel, Distribution of organelles and membranes between hepatocytes and nonhepatocytes in the rat liver parenchyma: A stereological study. J.Cell Biol. 72 (1977) 441-455.
